# Sintering Temperature, Frequency, and Temperature Dependent Dielectric Properties of Na_0.5_Sm_0.5_Cu_3_Ti_4_O_12_ Ceramics

**DOI:** 10.3390/ma14174805

**Published:** 2021-08-25

**Authors:** Hicham Mahfoz Kotb, Hassan A. Khater, Osama Saber, Mohamad M. Ahmad

**Affiliations:** 1 Department of Physics, College of Science, King Faisal University, P.O. Box 400, Al-Ahsa 31982, Saudi Arabia; hkhater@kfu.edu.sa (H.A.K.); osmohamed@kfu.edu.sa (O.S.); mmohamad@kfu.edu.sa (M.M.A.); 2 Physics Department, Faculty of Science, Assiut University, Assiut 71516, Egypt; 3 Egyptian Petroleum Research Institute, Nasr City, P.O. Box 11727, Cairo 11765, Egypt; 4 Department of Physics, Faculty of Science, The New Valley University, El-Kharga 72511, Egypt

**Keywords:** sintering, dielectric properties, impedance spectroscopy

## Abstract

NSCTO (Na_0.5_Sm_0.5_Cu_3_Ti_4_O_12_) ceramics have been prepared by reactive sintering solid-state reaction where the powder was prepared from the elemental oxides by mechanochemical milling followed by conventional sintering in the temperature range 1000–1100 °C. The influence of sintering temperature on the structural and dielectric properties was thoroughly studied. X-ray diffraction analysis (XRD) revealed the formation of the cubic NSCTO phase. By using the Williamson–Hall approach, the crystallite size and lattice strain were calculated. Scanning electron microscope (SEM) observations revealed that the grain size of NSCTO ceramics is slightly dependent on the sintering temperature where the average grain size increased from 1.91 ± 0.36 μm to 2.58 ± 0.89 μm with increasing sintering temperature from 1000 °C to 1100 °C. The ceramic sample sintered at 1025 °C showed the best compromise between colossal relative permittivity (ε′ = 1.34 × 10^3^) and low dielectric loss (tanδ = 0.043) values at 1.1 kHz and 300 K. The calculated activation energy for relaxation and conduction of NSCTO highlighted the important role of single and double ionized oxygen vacancies in these processes.

## 1. Introduction

Research for colossal permittivity materials (ε′ > 10^3^) with low dielectric loss (tanδ < 0.05) is an attractive topic because of the potential use of these materials in energy storage applications [1]. One of the most promising materials for such applications is CCTO (CaCu_3_Ti_4_O_12_) due to the stability of its colossal relative permittivity over a wide range of temperatures and frequencies [2,3]. Nevertheless, the dielectric loss of CCTO is considerably dependent on the preparation method [4,5,6]. The origin of the interesting dielectric properties of CCTO is controversially discussed in the literature. Several studies support extrinsic origins, such as the effects of sample/electrode barrier and internal barrier layer capacitors (IBLC) [7,8]. However, since CCTO single crystal also shows colossal permittivity [9], the intrinsic mechanisms cannot be neglected. Studies aiming at simplifying the fabrication process and reducing the dielectric loss at enough low level (tanδ < 0.05) while maintaining the colossal permittivity of titanate-based ceramics are still ongoing. These studies include the doping of CCTO to control the electrical properties of grain and grain-boundary of the ceramic [10,11,12,13,14], using simplified processes such as reactive sintering and sol-gel synthesis [12,15], and/or using of novel sintering techniques such as microwave sintering [4] and spark plasma sintering [6,13]. Na_0.5_Sm_0.5_Cu_3_Ti_4_O_12_ (NSCTO) ceramics showed interesting properties in terms of colossal relative permittivity (10^3^–10^5^) [16]. Recently, we reported on the dielectric properties of NSCTO prepared by reactive sintering solid-state reaction [15]. In this method, the starting raw materials are milled for several hours then directly sintered without passing by a calcination step. Both conventional sintering (CS) inside a tubular furnace in air (10 h at 1090 °C) and spark plasma sintering (SPS) (10 min at 1025 °C) under vacuum were studied. The reactively sintered NSCTO ceramics showed giant relative permittivity (ε′ > 10^3^) but high dielectric loss (tanδ > 0.08) at 1.1 kHz and 300 K [15]. In the current investigation, we report on the effect of the temperature of reactive CS inside a tubular furnace in air on the dielectric and structural properties of NSCTO ceramics. Phase purity and microstructure of the prepared ceramics have been monitored by X-ray diffraction (XRD) and field emission scanning electron microscope (FE-SEM) investigations. The dielectric spectra of the ceramics have been investigated in broad temperature and frequency ranges.

## 2. Materials and Methods

Stoichiometric amounts of Na_2_CO_3_ (99.99%, Aldrich, St. Louis, MO, USA), Sm_2_O_3_ (99.99%, Aldrich), CuO (99.995%, Aldrich), and TiO_2_ (99.9%, Aldrich) were firstly milled in Fritsch P-7 premium line machine for 10 h with a rotation speed of 500 rpm. Tungsten carbide pots and balls were used in the present study. Then, a suitable amount of the obtained powder was isostatically pressed (320 MPa) to pelletize the powder. The prepared pellets were sintered in air inside an electric tubular furnace at temperatures 1000 °C, 1025 °C, 1075 °C, and 1100 °C for 10 h. These samples will be abbreviated as CS-1000, CS-1025, CS-1075, and CS-1100. Morphology and phase characterizations were investigated by using field-emission scanning electron microscope (FE-SEM) (Joel, SM7600F, Tokyo, Japan) and powder X-ray diffraction (XRD) techniques. For XRD measurements, a Stoe Stadi-P Image Plate, IP, (Stoe and Cie GmbH, Darmstadt, Germany) Cu Kα1 radiation (λ = 1.5406 Å) was used. A turnkey concept 50 system from Novocontrol was used for impedance spectroscopy (IS) measurements in the frequency range of 1 Hz–40 MHz and temperature 120–400 K in dry nitrogen atmosphere. Sample temperature was automatically controlled by Quatro Cryosystem. Silver paint was applied on both sides of each pellet before the electrical measurements.

## 3. Results and Discussion

**Figure 1:**
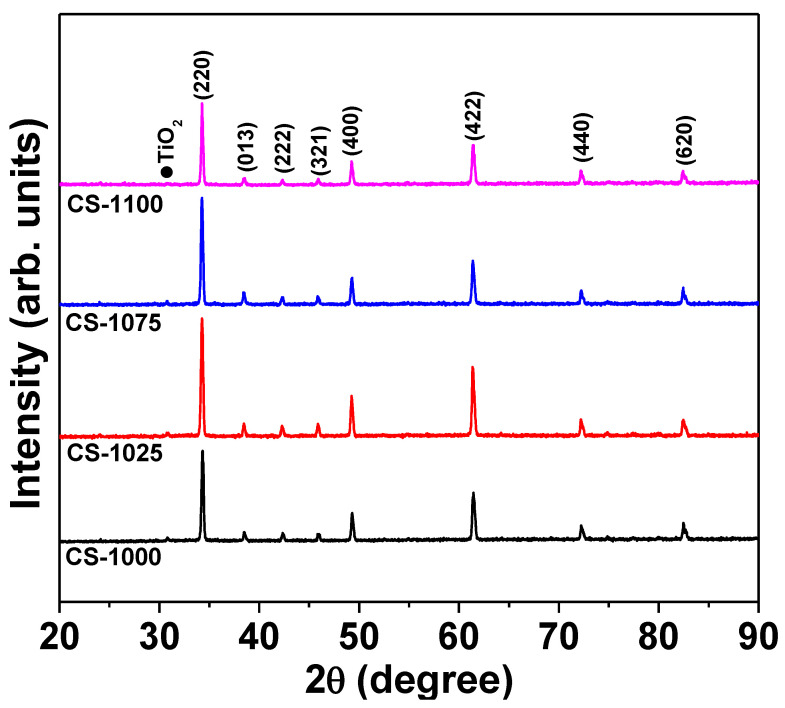
Room temperature XRD patterns of NSCTO ceramic samples.

Figure 1 shows the XRD patterns of the NSCTO ceramics. These patterns confirm the formation of CCTO-like cubic phase (JCPDS card no. 75-2188). A tiny peak located at 30.82° was observed for all the samples and could be attributed to the (400) crystal plan of TiO_2_ (JCPDS card no. 46-1238). By using the best-fit method (UnitCellWin software [17]), the lattice parameter was calculated from hkl and 2θ values of the XRD peaks. The lattice parameter for the current ceramics was found to be in the following range 7.391–7.395 Å, which agrees with the reported value of 7.394 Å for NSCTO ceramics [3,15].

In order to calculate the average crystallite size (*D*) and the lattice strain (*η*), the broadening of XRD peaks was analyzed according to the Williamson–Hall relation [18,19]:

(1) $$\beta \cos\theta =\frac{K\cdot \lambda }{D}+2\left(\eta \right)\sin\theta$$

where *β* is the diffraction peak’s width at half maximum (FWMH) in radian, *θ* is Bragg’s angle, *λ* = 1.5418 Å for the CuK_α_ radiation, and *K* is the shape factor (0.9). Therefore, by plotting (*β*cos*θ*) versus (sin*θ*), the values of *D* and *η* can be calculated from the intercept and the slope of the linear fit, respectively. Moreover, the dislocation density (*δ*) can be calculated from the *D* value by using Williamson–Smallman approach.

(2) $$\delta =1/D^{2}$$

The calculated values of *D*, *η*, and *δ* are summarized in Table 1. The positive value of *η* indicates a tensile strain in the current samples. The crystallite size increased from ~41 nm for the samples sintered at 1000–1075 °C to ~56 nm at a sintering temperature of 1100 °C. The increased crystallite size accompanied by the decrease in the strain and dislocation density indicated an improvement of samples’ crystallinity with sintering temperature.

**Figure 2:**
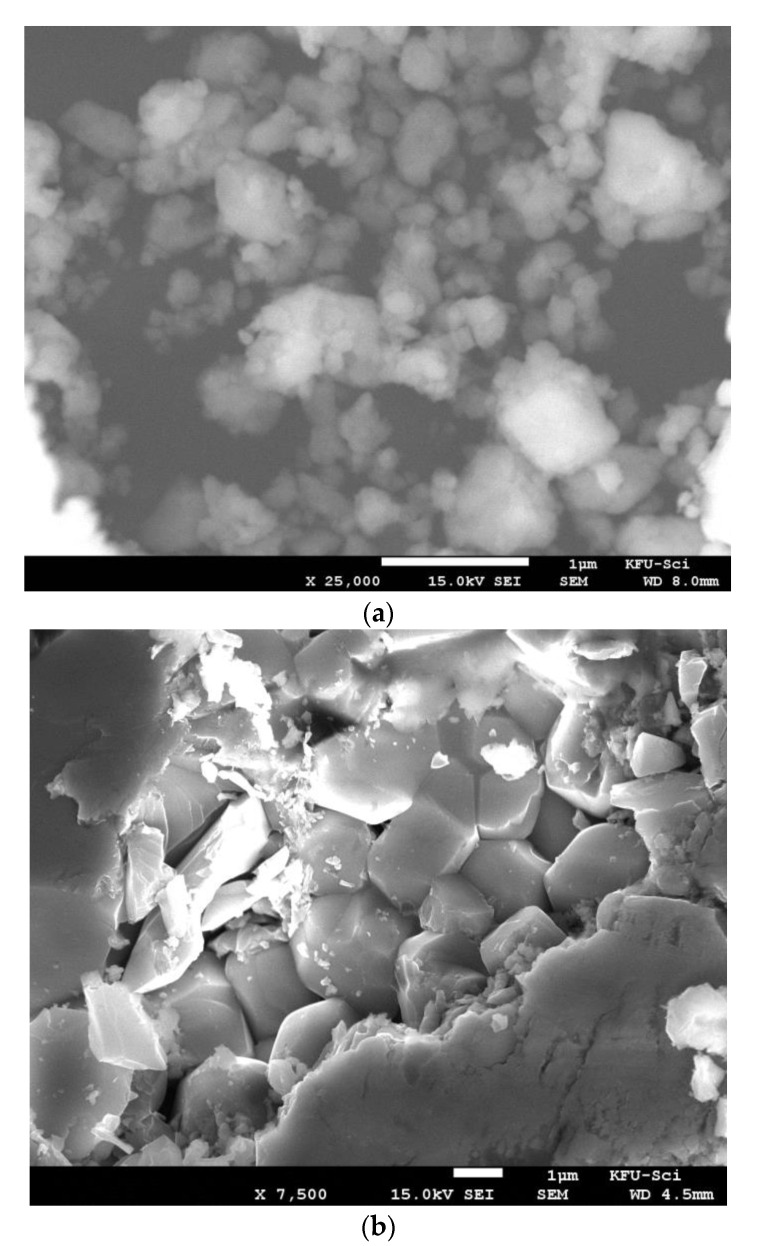

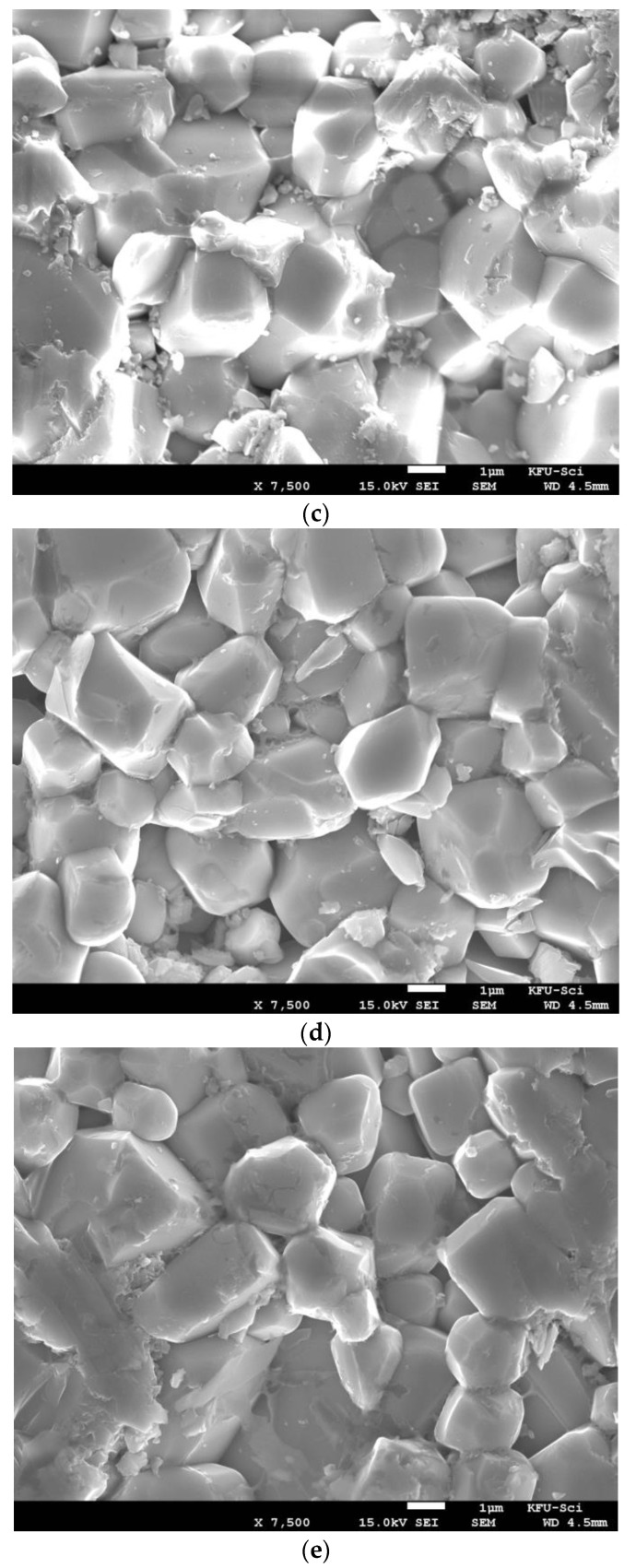
FE-SEM micrographs of (**a**) powder, (**b**) CS-1000, (**c**) CS-1025, (**d**) CS-1075, and (**e**) CS-1100 samples.

The morphology of the as-prepared powder and the sintered ceramics observed by FE-SEM are shown in Figure 2. The powder exhibited the formation of micrometric clusters or agglomerates which consist of finer particles. Moreover, the sintered samples revealed dense packed grains with low porosity, particularly for the sintering temperatures higher than 1000 °C. The average grain sizes determined by the linear intercept method [20] were found to be 1.91 ± 0.36 μm, 2.23 ± 0.56 μm, 2.34 ± 0.68 μm, and 2.58 ± 0.89 μm for CS-1000, CS-1025, CS-1075, and CS-1100, respectively.

**Figure 3:**
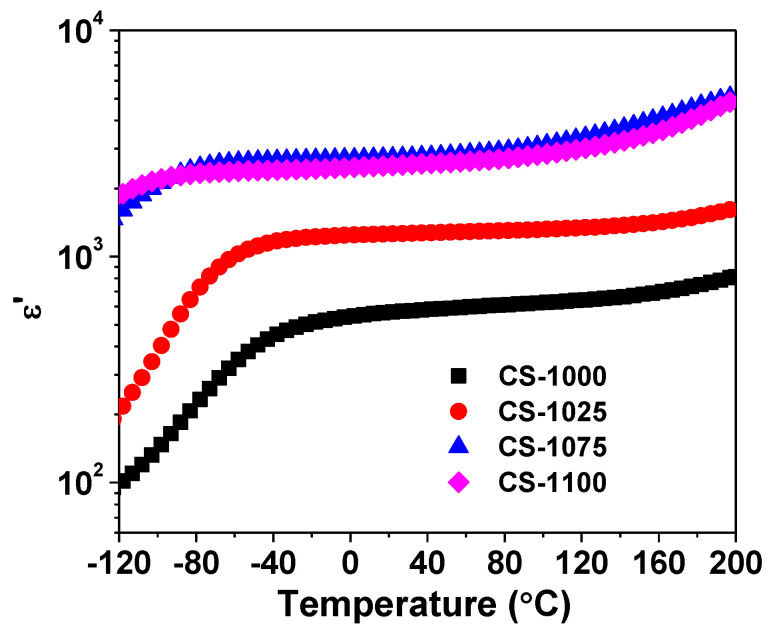
Temperature dependency of relative permittivity (ε′) at 10 kHz for the NSCTO ceramics.

**Figure 4:**
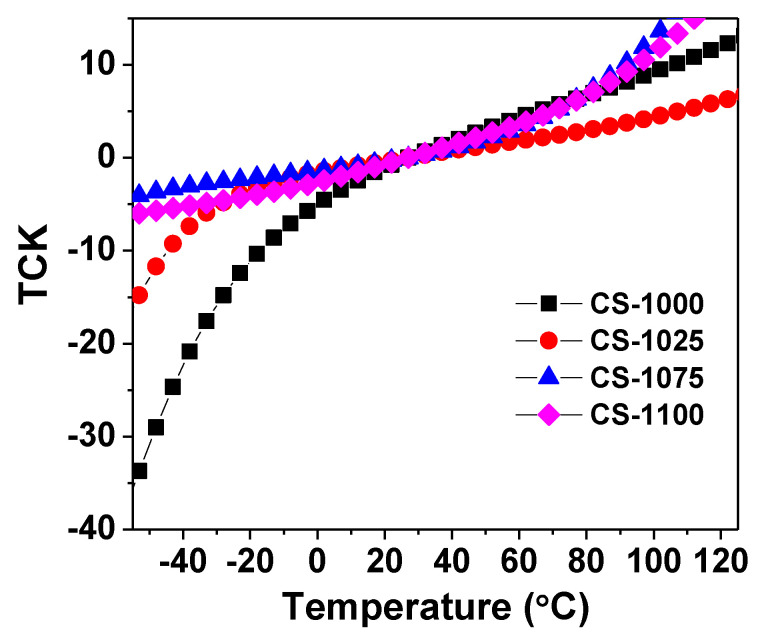
Thermal coefficient of permittivity as a function of temperature for the NSCTO ceramics.

Figure 3 reveals the temperature dependency of ε′ at 10 kHz for the NSCTO ceramics. As observed in this figure, ε′ of all ceramics is almost temperature-independent over a broad measuring temperature range from −50 °C to 120 °C. Moreover, ε′ increases with increasing sintering temperature. The ceramics CS-1075 and CS-1100 showed the highest ε′ (>10^3^) over the entire temperature range. It is known that temperature stability of permittivity is an important quality factor and can be evaluated by the thermal coefficient of permittivity (TCK) [21,22]

(3) $$TCK=\left(\frac{\Delta C_{T}}{C_{25^{\circ }C}}\right)=\left(\frac{C_{T}-C_{25^{\circ }C}}{C_{25^{\circ }C}}\right)\times 100\%$$

where *C_T_* is the sample capacitance at a given temperature and *C*_25°C_ is the sample capacitance at 25 °C. Figure 4 depicts the temperature dependence of *TCK* value of the current NSCTO ceramics at 10 kHz. Except for CS-1000, all ceramics showed good *TCK* values <±15% between −55 °C and 120 °C. The ceramic sample CS-1025 exhibited the best *TCK* values (−7.5 to + 5%) in the temperature range −40 °C to 120 °C. These findings indicate that NSCTO ceramics have good thermal stability as compared to the literature [22,23].

**Figure 5:**
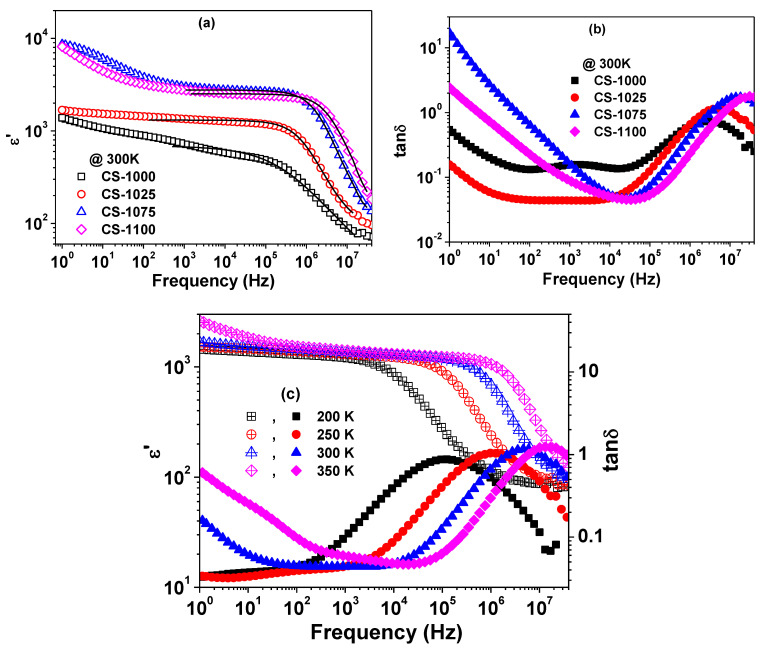
Frequency dependence of (**a**) *ε*′, (**b**) tanδ at 300 K for NSCTO ceramics, and (**c**) the frequency dependence of *ε*′ and tanδ at selected temperatures for CS-1025.

Figure 5 shows the frequency dependence of ε′ and tanδ for the NSCTO ceramics at room temperature. A plateau is observed in the spectra of ε′ followed by a step-like decrease with increase in frequency accompanied with a peak in the spectra of tanδ. The peak position shifted to higher frequency with increasing measuring temperature as shown in Figure 5c for CS-1025 as an example. This dielectric behaviour is reported elsewhere as a Debye-like relaxation process [24,25]. It is observed from Figure 5a,b that ε′ increased while the minimum value of dielectric loss tanδ)_min_ decreased with increasing sintering temperature. The room temperature value of ε′ at 1 kHz was found to be 717, 1344, 2982, and 2758 for NSCTO ceramics with increasing sintering temperature. The sample CS-1025 showed the lowest tanδ (~0.043) compared to the other samples with the additional advantages of having low tanδ values in a wide range of frequency (~10–10^4^ Hz) and better stability of ε′ over the frequency range 1–10^5^ Hz.

**Figure 6:**
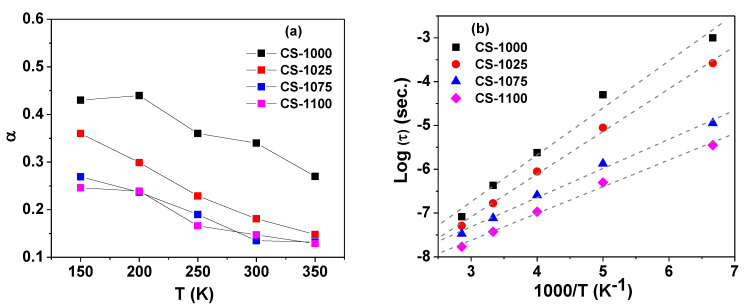
(**a**) The variation of the fitting parameter α with temperature, and (**b**) the Arrhenius plot for relaxation time for the NSCTO ceramic samples.

Moreover, the spectra of ε′ was found to fit properly with the modified Debye relationship [26]:

(4) $$\varepsilon^{*}=\varepsilon^{\prime }-i\varepsilon^{\prime \prime }=\varepsilon_{\infty }+\left(\varepsilon_{s}-\varepsilon_{\infty }\right)/\left[1+{\left(i\omega \tau \right)}^{1-\propto }\right]$$

where *ε_s_* and *ε*_∞_ are the static and high frequency relative permittivity, respectively, and *ω* is the angular frequency. The fitting parameters *τ* and *α* (0 < *α* ≤ 1) represent the relaxation time and the degree of the distribution of relaxation time, respectively, with *α* = 1 for an ideal Debye relaxation. The variation of fitting parameters with measuring temperature for the current ceramics is given in Figure 6a. The parameter α was found to be in the range 0.1–0.45, clearly indicating a multidispersive relaxation nature for the studied ceramics. As shown in Figure 6b, the temperature dependence of *τ* was found to obey the Arrhenius law [27]:

(5) $$\tau =\tau_{0}\exp\left(\frac{E_{R}}{k_{B}T}\right)$$

where *τ*_0_ is the pre-exponential factor and *E_R_* is the activation energy for the relaxation.

**Figure 7:**
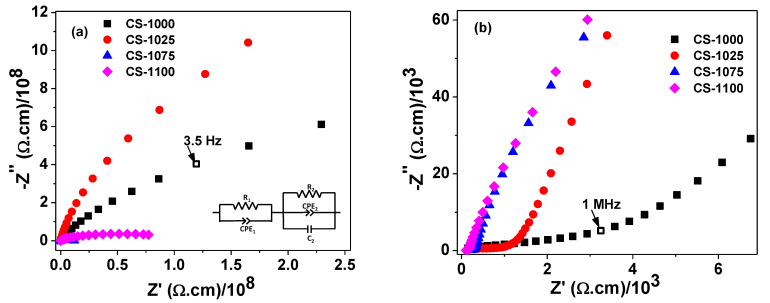
(**a**) Room temperature impedance complex plane plots for the NSCTO and (**b**) zoom-in for the high frequency region.

It is known that both ε′ and tanδ are closely related to the resistivity of the grain and grain-boundary of the sample. Therefore, the electrical properties of the ceramics were investigated by using the impedance spectroscopy measurements to differentiate the grain and grain-boundary resistivities. As observed in Figure 7a,b, at a given temperature, the complex resistivity plan plot for NSCTO ceramics is composed of two semi-circular arcs. The high frequency arc was assigned to the response of grain while the low frequency one was assigned to the grain-boundary response. The resistivity values of the grain and grain-boundary were calculated using the ZsimpWin 3.10 software (v3.10, Ametek, EChem software, Ann Arbor, MI, USA). The equivalent circuit that best fitted to the measured data of the complex impedance is shown in the inset of Figure 7a. The circuit symbols R, C, and Q represent the resistance, capacitance, and constant phase element (CPE) [28]. The calculated resistivity values of grain and grain-boundary are given in Table 2. As observed in this table, all NSCTO ceramics are electrically heterogeneous where the grain-boundary resistivity is several orders of magnitude higher than the resistivity of grain. This structure suggests the internal barrier layer capacitance (IBLC) effect [7,8] to explain the colossal dielectric constant of the current samples. This effect is based on Maxwell–Wagner polarization at the grain–grain-boundary interfaces due to the difference in their resistivities. According to the IBLC model, the effective dielectric constant is proportional to the grain size of the ceramic [29]. Hence the increase in ε′ with increasing sintering temperature is thought to be due to the increase in grain size as revealed by SEM observations. Moreover, the sample CS-1025 showed the highest ratio of R_g.b._/R_g_ (~7376), which might explain its low dielectric loss in the frequency range (~10–10^4^).

**Figure 8:**
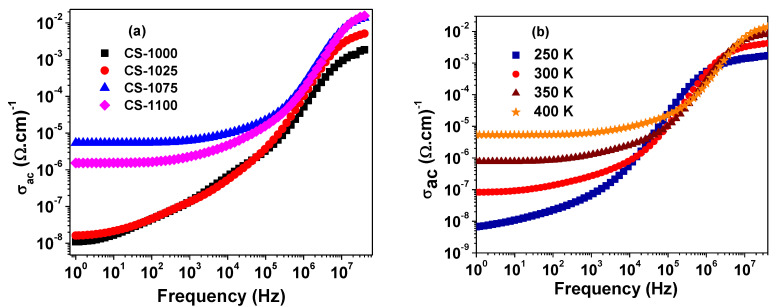
(**a**) Frequency dependency of *σ_ac_* at 400 K for NSCTO samples and (**b**) frequency dependency of *σ_ac_* at selected temperatures for CS-1075 NSCTO sample.

**Figure 9:**
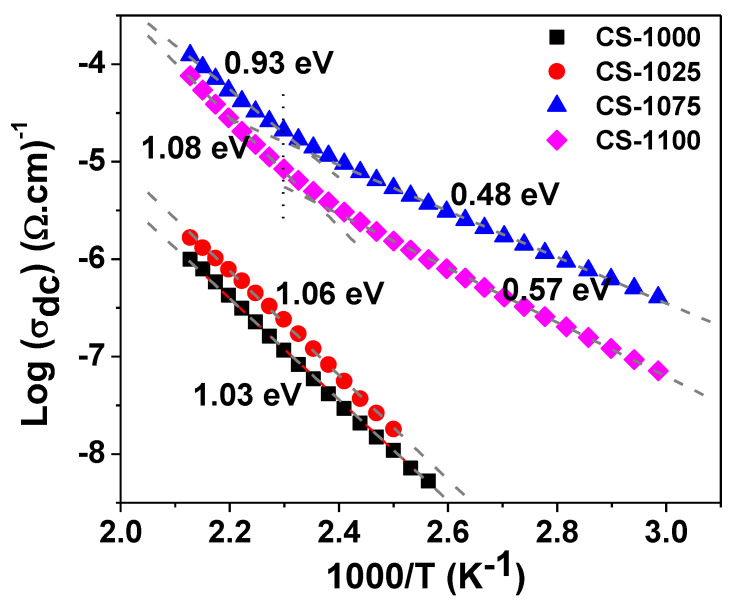
The Arrhenius plots of dc conductivity for CS-1000, CS-1025, CS-1075, and CS-1100 NSCTO samples.

Figure 8a depicts the spectrum of ac conductivity (*σ_ac_*) at room temperature for each of the sintered NSCTO ceramics. Two regions can be distinguished in each plot: one at the lower frequency side where *σ_ac_* tends to form a frequency independent plateau corresponding to the dc conductivity (*σ_dc_*) of the sample. With increasing frequency, in the second region *σ_ac_* becomes strongly dependent on the frequency, thus forming a dispersion region. The conductivity increases, and the plateau region becomes wider with increasing temperature, as can be observed in Figure 8b for CS-1075 as an example. Figure 9 shows the dependence of log(*σ_dc_*) on the reciprocal of temperature for the current ceramics according to the following Arrhenius relationship:

(6) $$\sigma_{dc}=\sigma_{0}\exp\left(\frac{-\Delta E}{k_{B}T}\right)$$

where *σ*_0_ is the pre-exponential factor, *k_B_* is the Boltzmann constant, and ∆*E* is the activation energy for conduction. The obtained good linear fit in Figure 9 indicates the nearest neighbor hopping (NNH) as the predominant conduction mechanism in NSCTO ceramics. The calculated values of the activation energy for conduction are summarized in Table 2.

**Figure 10:**
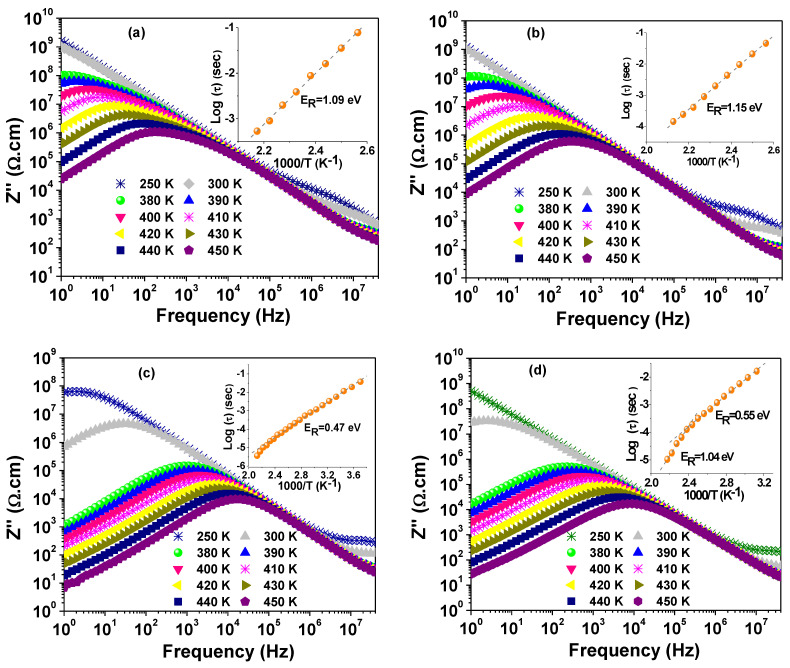
Spectra of Z″ at selected temperatures for (**a**) CS-1000, (**b**) CS-1025, (**c**) CS-1075, and (**d**) CS-1100. The inset of each figure depicts the Arrhenius plot for the relaxation time.

Figure 10 illustrates the evolution of the frequency response of Z″ with increasing measuring temperature for NSCTO ceramics. Two relaxation peaks can be observed in the spectra. One peak with low intensity appears at low temperature and high frequency side, while the other peak with considerably higher intensity appears at higher temperature and low frequency. For both peaks, the peak maximum decreased, and the corresponding peak frequency (f_max_) shifted towards higher frequency with increasing temperature. Moreover, the peak maximum of the low frequency peak is several orders of magnitude higher than the high frequency one. This implies that the low and high frequency peaks correspond to grain-boundary and grain responses, respectively. The relaxation time (*τ* = 1/2πf_max_) was calculated for the well resolved low frequency peak (grain-boundary response), and the dependence of log(*τ*) on the inverse of temperature was given in the insets of Figure 10. It is implied from these plots that the behaviour of the NSCTO ceramics obeys the following Arrhenius law:

(7) $$\tau =\tau_{0}\exp\left(\frac{E_{R}}{k_{B}T}\right)$$

where *τ*_0_ is the pre-exponential factor, *E_R_* is the activation energy for conductivity relaxation, and *k_B_* is Boltzmann constant. Nevertheless, as can be observed in the inset plots for CS-1075 and CS-1100, a deviation takes place at temperatures higher than ~390 K where a second linear region is formed. This deviation is more obvious in the plot for CS-1100. The calculated values of *E_R_* from the slopes of the linear regions were added to Table 2.

It is known that oxygen vacancies develop in perovskite materials during high temperature treatments; therefore, they act as mobile charge carriers in these materials [30]. Moreover, the ionization of oxygen vacancies creates free conducting electrons. The possible reactions can be defined by Kroger–Vink notation as follows:

(8) $$O_{O}\Leftrightarrow V_{O}+1/2O_{2}$$

(9) $$V_{O}\Leftrightarrow V_{O}^{•}+e^{\prime }$$

(10) $$V_{O}^{•}\Leftrightarrow V_{O}^{••}+e^{\prime }$$

where $V_{O}^{•}$ and $V_{O}^{••}$ represent single and double ionized oxygen vacancies. By cooling down of the sample, reoxidation takes place at the grain-boundary and the surface of grains, which results in higher resistivity of the grain-boundary. Hence, the higher resistivity of CS-1000 and CS-1025 is thought to be due to the lower content of oxygen vacancies and therefore lower content of free electrons in these samples. It was reported that oxygen vacancies have activation energy in the range 0.1–0.5 eV and 0.6–1.2 eV for singly and doubly ionized oxygen vacancies, respectively [30,31,32]. Therefore, the findings of the current study suggest that doubly ionized oxygen vacancies predominate the conduction and relaxation processes in the grain boundaries of NSCTO. On the other hand, singly ionized oxygen vacancies dominate the conduction and relaxation processes in grains. Moreover, the systematic decrease in the grain resistivity with sintering temperature indicates the correlated increase in oxygen vacancy contents with sintering temperature.

## 4. Conclusions

Na_0.5_Sm_0.5_Cu_3_Ti_4_O_12_ ceramics with colossal permittivity and low dielectric loss were prepared by a simplified solid-state reaction. The sintering temperature varied between 1000 and 1100 °C for 10 h. XRD analysis confirmed the formation of almost pure cubic phase for all samples. In the sintering temperature range 1000–1075 °C, the average crystallite size, lattice strain, and dislocation density were found to vary slightly while a considerable change in these quantities took place after sintering at 1100 °C. SEM observations revealed the increase in grain size of NSCTO ceramics from 1.91 ± 0.36 μm to 2.58 ± 0.89 μm with increasing sintering temperature from 1000 °C to 1100 °C. The ceramic sample sintered at 1025 °C showed the best compromise between colossal relative permittivity (ε′ = 1.34 × 10^3^) and low dielectric loss (tanδ = 0.043) values at 1.1 kHz and 300 K. On one hand, the activation energy for dielectric relaxation in grains was found to decrease considerably from 0.212 eV to 0.121 eV with increasing sintering temperature. On the other hand, the activation energy for dielectric relaxation in grain-boundaries was found to be in the range 0.48–1.08 eV without systematic behaviour. These findings support the conclusion that the conduction and dielectric relaxation processes in NSCTO are dominated by the movement of single and double ionized oxygen vacancies. The colossal relative permittivity, low dielectric loss, and good temperature stability of the dielectric properties of the studied NSCTO ceramics recommend their suitability for the energy storage applications.

## Figures and Tables

**Table 1:** The lattice parameter (c), the average crystallite size (*D*), lattice strain (*η*), and dislocation density (*δ*) of NSCTO ceramics.

Sample	c (nm)	*D* (nm)	*η* ×10^−3^	*δ* ×10^14^ (Line/m^2^)
CS-1000	0.7391	40.57	1.11	6.07
CS-1025	0.7397	41.17	0.99	5.89
CS-1075	0.7396	41.05	1.03	5.93
CS-1100	0.7395	55.95	1.94	3.19

**Table 2:** The resistivity of grain (R_g_), grain-boundary (R_g.b._), activation energy for conduction (ΔE), and for relaxation process (E_R_) in NSCTO ceramics.

	R_g_ (Ω.cm)	R_g.b._ (Ω.cm)	Δ*E* (eV)	*E_R_* (eV) Grain	*E_R_* (eV) Grain-Boundary
CS-1000	4601	3.15 × 10^9^	1.03	0.212	1.15
CS-1025	1169	8.623 × 10^9^	1.06	0.193	1.09
CS-1075	332	1.30 × 10^7^	0.48 (T < 390 K) 0.93 (T > 390 K)	0.132	0.47 (T < 390 K)
CS-1100	219	1.09 × 10^8^	0.57 (T < 390 K) 1.08 (T > 390 K)	0.121	0.55 (T < 390 K) 1.04 (T > 390 K)

## Data Availability

Not applicable.
